# Cardioprotective function of mixed spices against myocardial infarction injury: In-vivo and in-silico study

**DOI:** 10.1016/j.jgeb.2025.100492

**Published:** 2025-04-19

**Authors:** Md. Abdullah-Al-Mamun, Dipa Islam, Dipankar Chandra Roy, Ayesha Ashraf, Saifullah, Chadni Lyzu, Md. Enamul Kabir Talukder, Samina Akhter, Evana Parvin Lipy, Liton Chandra Mohanta

**Affiliations:** a Bangladesh Jute Research Institute, Dhaka-1207, Bangladesh; b Biomedical and Toxicological Research Institute, Bangladesh Council of Scientific and Industrial Research, Dhaka-1205, Bangladesh; c Biotechnology and Genetic Engineering Discipline, Life Science School, Khulna University, Khulna 9208, Bangladesh; d Department of Genetic Engineering and Biotechnology, Faculty of Biological Sciences and Technology, Jashore University of Science and Technology, Jashore-7408, Bangladesh

**Keywords:** Myocardial Infarction, Mixed spices, Isoproterenol, Caspase-3, CADD

## Abstract

Myocardial infarction is the permanent necrosis of heart tissue caused by an artery obstruction. It causes an inadequate delivery of oxygen and nutrients, resulting in muscle injury in the afflicted areas. Here, water extract of mixed spices—onion, garlic, ginger, red chili, turmeric, cumin seed, coriander, cardamom, black pepper, cloves, fenugreek, nigella, cinnamon, and carom seed—was prepared to evaluate cardioprotective function in albino rats. To systematically investigate cardioprotective efficacy, isoproterenol was injected into albino rats to induce myocardial injury. The prepared extract was administrated orally to rats daily for 28 days (200 mg/kg body weight) before infusion of isoproterenol (100 mg/kg body weight) on 29th and 30th days. The induced cardiac injury was significantly ameliorated in rats based on cardiac hypertrophy, histopathology, and *Caspase-3* mRNA expression analysis by qRT-PCR. The Indian Medicinal Plants, Phytochemistry And Therapeutics (IMPPAT) chemical database of 820 natural compounds from the mixed spices was then screened against CASP-3 protein using cheminformatics tools, where thymohydroquinone, 4-isopropylbenzoic acid, and 1-naphthylacetic acid were found to be the best interacting ligands, with binding energy scores of −6.112 kcal/mol, −6.206 kcal/mol, and −6.112 kcal/mol, respectively. Notably, thymohydroquinone exhibited the lowest predicted cytotoxicity. Furthermore, molecular dynamic simulation was used to validate the binding stability of the thymohydroquinone with CASP-3 protein compared to CID-6167 (control). Thus, this study explored that mixed spices have cardioprotective effects in rats and identified thymohydroquinone as a natural lead compound against CASP-3, which may pave the way for the development of pharmacotherapy for myocardial damage.

## Highlights

• Spices Mix attenuated ISO-induced cardiac hypertrophy and tissue damage, confirmed by histology and morphometry.
• Spices Mix downregulated Caspase3 gene expression, reducing myocardial apoptosis in ISO-treated rats.
• In-silico analysis identified thymohydroquinone as a potent CASP-3 inhibitor, offering therapeutic potential.
• In-vivo and in-silico validation confirms the spice mix's cardioprotective effects.

## Graphical abstract

**Figure:**
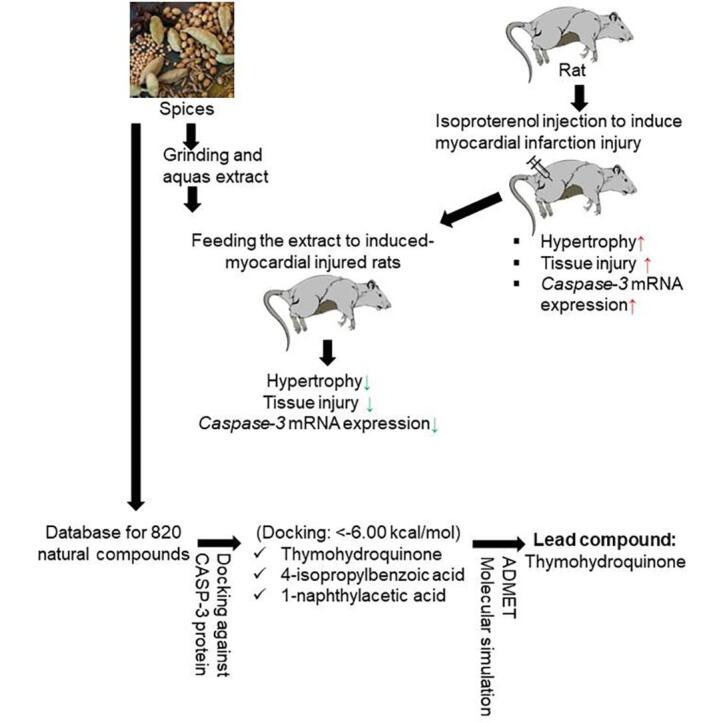

## 1 Introduction

Myocardial Infarction (MI) is a multifactorial, polygenic condition that is the main cause of mortality in developing and industrialized nations.^1^, ^2^ It's necrosis of cardiac muscle from ischemia.^3^ MI's pathophysiology includes cholesterol deposits in the subendothelial vascular intima, artery wall inflammation, plaque rupture, fissure, or erosion, and blood clot development.^4^ MI frequency rises with age and is regulated by environmental and genetic variables.^5^ Epigenetic processes govern pathogenic pathways due to gene-environment interactions. Epigenetic modifications impact gene transcription without altering DNA sequence.^6^.

Apoptosis contributes to cell death in a regulated pathway^7^ and it depends on cascade-activated intracellular proteases called caspases.^8^ The 'initiator' caspases-8 and caspase-9, and the 'effector' caspase-3 play key roles in apoptosis^9^ and heart failure in both experimental animals and humans.^10^ Caspase3 is activated in end-stage heart failure patients' myocardium and overexpressed in right ventricular dysplasia patients' myocardium,^11^ showing its significance to heart disease.

The growing number of MI patients worldwide requires improved preventative and treatment measures. Western medicine is popular. Antithrombotic medications coupled with reperfusion treatment, CABG, or PCI are frequently used for CVD (Cardiovascular Disease) patients.^12^ Although these treatment regimens reduce cardiovascular events, significant side effects remain a barrier.^13^ Thus, alternative and complementary medicines were sought to treat CVDs. Polyphenols from spices including curcumin from turmeric,^14^ 6-shogaol from ginger,^62^ trigonelline from fenugreek,^63^ garlic supplements,^15^ nigella supplements^64^ and Ginseng, *Ginkgo biloba*, *Ganoderma lucidum*, and *Gynostemma pentaphyllum*^16^ help protect against cardiovascular problems. Coriander, cardamom and cumin seed have also positive cardiovascular effects.^65^, ^66^, ^67^ With the efficacy of Chinese Herbal Medicine (CHM) in CVD prevention and treatment, its effects have gained increased attention, especially in industrialized nations like the U.S. and Australia.^12^ Moreover, mixed spices constitute a combination of different spices that have different biological significances. Animal trials of a mixture of nigella and ginger with chicory and gurmar have ensured positivity in hypertension treatment.^68^ Culinary doses of mixed spices have potential prebiotic effects,^69^ antioxidant and antimicrobial effects,^71^ and can improve blood pressure.^70^ Cellular study of a mixture of turmeric, garlic and ginger has demonstrated the therapeutic potentiality of treating cancer.^72^ Also, different spices mixture has digestive stimulatory effects^73^ as well as hepato- and cardio-protective effects.^74^.

Computer-aided drug design (CADD) has been used for more than thirty years to screen, develop, and generate therapeutically significant molecular candidates.^17^ These techniques combine ADME (absorption, distribution, metabolism, and excretion), toxicity, molecular docking, and molecular dynamics (MD) simulation to optimize and test drugs. Recently, molecular docking has been a prominent approach for determining target protein-small molecule interactions.^18^ The approaches can suggest promising compounds for drug design, and MD simulation may confirm their stability to the target protein.^19^ The ADME emphasizes pharmacokinetics and pharmacology elements to design drugs and helps us to evaluate a small molecule candidate's safety and efficacy. Drug design may also include computational toxicity research to evaluate a chemical's biological effects or a tiny molecular contender.^20^ CADD techniques have found several small molecular candidates that have become innovative in cardiovascular disease treatments trough passing clinical trials.^21^.

Therefore, the study aimed to examine the cardio-protection impact of various spices in Winstar albino rat hearts following isoproterenol-induced myocardial damage utilizing anthropometric, histological, and *Caspase-3* mRNA expression studies as well as to identify bioactive natural compounds of fourteen mixed spices through molecular docking, ADME, toxicity and finally molecular dynamics simulation approaches identified the lead compound as an alternative treatment for myocardial infarction.

## 2 Materials and methods

### 2.1 In-Vivo experiment

#### 2.1.1 Experimental animals

Animals were chosen, housed, fed, and prepared for the experiment following OECD criteria.^22^ Biomedical and Toxicological Research Institute (BTRI), Bangladesh Council of Scientific and Industrial Research (BCSIR), provided 24 developing Wister albino rats (10–14 weeks, 160–240 g) which were randomly separated into four groups (each group comprised male and female rats) for a 30-day study. Animals were acclimatized to laboratory settings for a week before experiments, kept in rectangular cages made of polypropylene (50–35-20 cm) with a 12/12 h light/dark photoperiodic cycle, 23 ± 58 °C temperature, and 40–70 % dryness after reducing non-specific stress. An ethical committee approved all experiments. Rat meal was 100 % lab food (Supplementary file-1).

#### 2.1.2 Spices Mix and extract Preparation, ISO dosages and MI induction

Fourteen types of raw spices were purchased from Dhaka New-market, Bangladesh. The spices were authenticated by scientific officer (Botany), BCSIR. After washing and sun-drying, electric blenders (YT-4677A-S Miyako, Japan) were used to powder the spices. Spices mixture (SM) was prepared in a composition (Supplementary file-1) and prepared aqueous extract using the protocol in *protocol.io*.^23^ SM was diluted with 2 ml distilled water for each rat (200 mg/kg body weight) to prepare spices mix doses. Lab diet and spice mixture were given orally before the sacrifice. ISO powder (Tokyo Chemical Industry Ltd., Japan) was diluted with 0.9 % NaCl at 85 mg/kg concentration in distilled water.^24^ ISO solution was injected twice, 24 h apart, to induce MI.

#### 2.1.3 Experimental design

Twenty-four rats of equal weight were randomized into four groups and fed varied diets. All rats received lab food and varied treatment dosages: Untreated Control (UTC) group: Distilled water (consecutive 28 days) + Normal saline (29th and 30th day). Isoproterenol (ISO) group: Distilled water (consecutive 28 days) + ISO solution (29th and 30th day) at a dose of 100 mg/kg/day. Mixed spices group: Spices mix (consecutive 28 days) at a dose of 200 mg/kg/day + Normal saline (29th and 30th day). ISO + Mixed spices group: Spices mix (consecutive 28 days) at a dose of 200 mg/kg/day + ISO solution (29th and 30th day) at a dose of 100 mg/kg/day.

#### 2.1.4 Weight and length measurement of rats

On day one, rats' weights and lengths were measured. Each rat's weight was measured by placing it in a plastic container on a digital scale. Skilled workers straightened each rat on a sterilized table. The 0-end of a measuring scale was inserted in the rats' tails, while the other end was held in their noses. After the first trial day, weight and length were measured every seven days till rat slaughter (data are in Supplementary file-2). Weight gain and BMI (Body Mass Index) of animals were calculated from the obtained data using the following formulas:

$$\mathrm{Weight}\;\mathrm{gain}=\left(\mathrm{Final}\;\mathrm{Body}\;\mathrm{Weight}-Initial\;Body\;Weight\right)$$

$$\mathrm{BMI}=Body\;weight÷{\left(\mathrm{Length}\right)}^{2}$$

#### 2.1.5 Sacrifice of animals and organ collection

To sacrifice, rats were anesthetized within 24 h of last ISO administration with 0.5 mL/rat intraperitoneal injection of G-Ketamine (conc. 50 mg/ml) (Gonoshasthaya Pharmaceuticals Limited, Dhaka, Bangladesh) for lowered blood pressure and zero movements. Anesthetized rats were kept on wax trays in the OT lab and legs were stretched, connected to pins, and incised neck-to-belly. The heart, liver, and kidney were excised. After squeezing blood in ice-cold saline, incised organs were cleaned and weighed to measure organ weight to body weight (data are in Supplementary file-2) where heart weight to body weight is defined as cardiac hypertrophy. One section of incised tissue was soaked in 10 % formalin buffer (Merck, Germany) at room temperature for seven days for histopathological research and the other in 0.9 % NaCl keeping at −80 °C for molecular analysis.

#### 2.1.6 Histopathological analysis

Histopathological analysis was performed according to Islam et al., with slight modifications.^75^ After soaking in acetone (20 min, 150 rpm) and xylene (30 min, 150 rpm), tissues were promptly immersed in paraffin and incubated (71 °C, 20 min) to make FFPE tissue embedding system. Next, paraffinized tissue blocks were made in an embedding unit (Sakura TEK5, Japan) and maintained at −20 °C. Slices (3 μm) were cut in a microtome machine (MR 2258, Italy) and fixed on histology slides, then deparaffinized (60 °C, 1 h). Histological slides were stained with Haematoxylin and Eosin (H&E) and seen using a fluorescence microscope (Olympus BX 43, Tokyo, Japan) at 20 magnifications. A digital camera was used to take photomicrographs.

#### 2.1.7 Total RNA isolation

Total RNA was isolated from heart, liver, and kidney tissues using SV Total RNA Isolation System (Promega Corporation, Madison, WI, USA) with modification of the manufacturer’s protocol.^25^ Briefly, the fresh tissue or frozen tissue samples were lysed by the addition of lysis buffer (RLA), and homogenized by vortexing for 2–3 min. Next, 350 µL/tube dilution solution (RDA) was added and mixed by inversion four times. The sample was centrifuged at 20,000 × g for 10 min at 4 °C, and the supernatant was shifted to a new tube. Next, 200 µL of 100 % ice-cooled ethanol was added to the supernatant and mixed by pipetting. The sample was then transferred to the Spin Basket Assembly column, and centrifuged at 8,000 × g for 15 s, and the supernatant was discarded. The column was washed twice with 600 µL of wash buffer (RWA), and the RNA was eluted into 50 µL of RNAase-free water. The eluted RNA was stored at a −80 °C freezer for use in gene expression study.

#### 2.1.8 SYBR green-based RT-qPCR

GoTaq ® 1-Step RT-qPCR System (Promega Corporation, Madison, WI, USA) was used for SYBR Green-based qPCR in a 10 μL volume on a QuantStudioTM 6 Flex Real-Time PCR System (Applied BiosystemsTM, USA) according to our prior technique.^26^ The reaction consists of 3 µL water, 5 µL of GoTaq® qPCR master Mix, 0.2 μL of 50 × GoScript^TM^ RT Mix, 1 μL RNA template (100 ng), 0.4 μL of each primer. The primer sequences and reaction conditions are provided in Supplementary file-1. The C_t_ values were normalized against *B2M* gene using the 2^-ΔΔCT^ method.^27^, ^28^.

#### 2.1.9 Statistical analysis

The statistics are mean ± SEM (Standard error of the mean). We utilized *t*-test to compare group means. A one-way ANOVA were used for analyzing all of the study’s data. 0.05 was judged significant. All graphs were made using GraphPad Prism 8 and PowerPoint (2016).

### 2.2 In-Silico study

#### 2.2.1 Retrieval and preparation of protein structure

The 3D X-ray crystallographic structure of the caspase-3 (PDB: 3KJF) was repossessed from the RCSB-PDB.^29^ The resolution of the structure was determined to be 2.00 Å. In the protein preparation process using the Schrodinger suite version 2020–3, various steps were taken to optimize the protein.^29^ Bond orders were assigned, and zero-order bonds were created for metals. Disulphide bonds were created to fill any missing side chains and water molecules were removed from the protein. To further refine and minimize the energy of the protein crystal structure, the Optimized Potential for Liquid Simulations (OPLS-3e) force field was used.^32^.

#### 2.2.2 Preparation of ligands

Natural phytochemicals from medicinal plants or spices provide varied chemical domains for drug design and discovery. The IMPPAT server, a manually maintained database, uses cheminformatics to improve natural product-based drug discovery.^30^ We annotated 820 distinct compounds from the database of 14 spices' phytochemicals in Supplementary file-3 that had been pharmacokinetics and toxicity profiled. These 820 pharmacologically active phytochemicals were retrieved for ligand preparation. The Ligprep wizard of the Maestro Schrodinger package processed.^29^, ^31^ Epik 5.3 minimized ligand molecule high-energy ionization states at pH 7.02. The OPLS3e force field identified chiral centres on each molecule and generated stereoisomers, followed by another minimization process.^32^ .

#### 2.2.3 ***Analysis of the protein***–***ligand binding score by molecular docking***

The prepared ligands from the 14 mixed spices docked with the caspase-3 protein (PDB:3KJF), the Glide package of the Glide v-8.8 and Maestro v-12.5.139 programs to assess and illustrate the best binding scores between phytochemicals and target protein. The OPLS3e force field is employed in the standard precision mode for docking. A grid box corresponding to full protein positions has been created and the dimensions of the grid box are 10 × 10 × 10 Å^3^.^33^ Then, SP molecular docking was conducted on caspase-3 proteins with selected phytochemicals and standard drugs. From these molecular docking, target proteins and ligand-binding energy were extracted.

#### 2.2.4 Analyses of pharmacokinetics, toxicity, physicochemical properties, drug-able properties, and synthetic accessibility

In the drug development process, the investigation of ADME/T, physicochemical properties, lipophilicity, water solubility, drug-likeness, and synthetic accessibility identifies compounds with the best pharmacokinetic characteristics and potential as useful druggable compounds.^34^ We conducted a study on the top three Phyto-ligand molecules with a negative highest docking score from the standard drugs. To predict the pharmacokinetics, physicochemical properties, lipophilicity, water solubility (Log S (ESOL)), drug-likeness and synthetic accessibility, we uploaded the SMILES format data of these molecules to the SwissADME server (https://www.swissadme.ch/).^34^ Moreover, to determine the toxicity of these molecules through a free web server called ProTox-II (https://tox-new.charite.de/).^35^.

#### 2.2.5 Molecular dynamics simulation

The complex structures were simulated for 100 ns using the “Desmond v6.3 Program” in Schrodinger 2020–3 under Linux to assess the protein–ligand interaction stability in the chosen candidate compounds. A TIP3P water model was used to simulate the caspase-3 and phytocompound combination. To maintain a particular volume, an orthorhombic box measuring 10 Å from the center was utilized. Na + and Cl- were injected to neutralize the system with a 0.15 M salt concentration.^36^ An OPLS3e force field was used. The protein–ligand complex system was reduced using a natural time and pressure (NPT) ensemble at 101,325 Pascals and 300 K. To assess complex stability and dynamics, RMSD, RMSF, Rg, and SASA values were examined.

## 3 Results

### 3.1 Anthropometric measurement

**Fig. 1:**
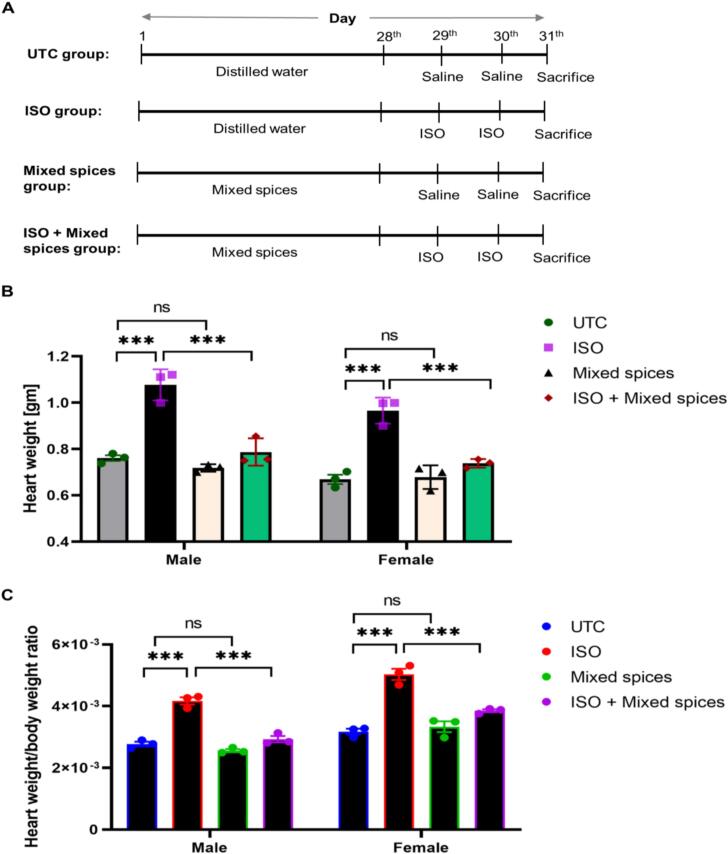
**Cardiac hypertrophy analysis after the mixed spices treatment.** A, Schematic diagram of experimental design. Twenty-four rats were classified into four groups, UTC, untreated control; ISO, isoproterenol-induced MI group rats; Mixed spices, mixed spices treated group rats to observe the inverse effect of the spices; and ISO + Mixed spices, the mixed spices treatment group rats. B, Heart weight measurement. Heart weight of ISO group was in the highest pick while reduced weight was depicted in ISO + Mixed spices group. Mixed spices had a little bit or no change in heart weight compared to UTC group. C, Heart weight/body weight ratio (cardiac hypertrophy). ISO groups’ ratio was highest among all groups. Approximately similar ratio was found in both Mixed spices and ISO + Mixed spices group compared to UTC group. All data represented here are triplicate. *** indicates the groups are statistically significant (p less than 0.05). “ns” indicates no significance.

At 31st day, animals were sacrificed after consuming distilled water (UTC and ISO group) and mixed spices solution (Mixed spices and ISO + Mixed spices group) at 29th and 30th day (Fig. 1A). After sacrifice, available and analysed data shows a significant difference (*p* = 0.05) between UTC and ISO groups as well as ISO and ISO + Mixed spices groups in heart weight and heart weight/body weight ratio (cardiac hypertrophy) for male and female rats. Comparing UTC and Mixed spices for heart weight and heart weight/body weight ratio, no significant difference (p < 0.05) was found (Fig. 1B and Fig. 1C). These findings show the most cardiac hypertrophy in the ISO group, a considerable improvement in the ISO + Mixed spices group, and no harmful impact on the heart in the Mixed spices group.

Table 1 shows no significant difference (p < 0.05) between male and female rat Kidney Weight (KW), kidney weight/final body weight (KW/FBW), Liver Weight (LW), and liver weight/FBW ratio. The ISO group exhibits a significant difference (p < 0.05) from the UTC group in male and female FBW, male weight increase, and female BMI, showing weight and BMI decrease.

**Table 1:** Changes of various anthropometric parameters for both male and female rats.

**Parameters**		**UTC**	**ISO**	**Mixed spices**	**ISO + Mixed spices**
Initial body weight (IBW)	M	224.33 ± 1.20	218.67 ± 0.67^a^	232.33 ± 0.67^a,b^	222.33 ± 1.33
	F	184.67 ± 0.88	172 ± 1.53^a^	180 ± 1^a,b^	172 ± 1.55^a^
Final body weight (FBW)	M	274.67 ± 3.18	258 ± 2^a^	280.67 ± 3.18^b^	268.33 ± 2.40^b^
	F	210.7 ± 2.40	192 ± 2.31^a^	205 ± 2.33^b^	191.67 ± 3.53^a^
Weight gain	M	50.33 ± 2.03	39.33 ± 1.76^a^	48.33 ± 2.6	46 ± 1.15^b^
	F	26 ± 1.73	20 ± 2.52	23.67 ± 1.45	19.67 ± 2.91
Initial BMI	M	0.46 ± 0.002	0.53 ± 0.04	0.48 ± 0.001^a^	0.49 ± 0.02
	F	0.48 ± 0.02	0.46 ± 0.01	0.47 ± 0.04	0.46 ± 0.01
Final BMI	M	0.54 ± 0.02	0.49 ± 0.03	0.53 ± 0.02	0.51 ± 0.005
	F	0.48 ± 0.005	0.41 ± 0.003^a^	0.45 ± 0.009^b^	0.44 ± 0.02
Kidney weight (KW)	M	1.22 ± 0.06	1.21 ± 0.07	1.13 ± 0.09	1.18 ± 0.12
	F	1.10 ± 0.1	1.15 ± 0.07	1.14 ± 0.1	1.11 ± 0.07
KW/FBW	M	0.004 ± 0.0002	0.005 ± 0.0003	0.004 ± 0.0003	0.004 ± 0.0005
	F	0.005 ± 0.0005	0.006 ± 0.0003	0.006 ± 0.0005	0.006 ± 0.0004
Liver weight (LW)	M	6.18 ± 0.23	6.31 ± 0.16	6.30 ± 0.19	6.05 ± 0.19
	F	6.02 ± 0.21	5.77 ± 0.19	6.04 ± 0.35	5.96 ± 0.09
LW/FBW	M	0.02 ± 0.001	0.02 ± 0.0004	0.02 ± 0.0009	0.02 ± 0.0008
	F	0.03 ± 0.001	0.03 ± 0.0007	0.03 ± 0.001	0.03 ± 0.0001

**Legends:** Results are expressed as Mean ± SE; n = 06. a,b Values in the same row that do not share superscript letters (a, b) differ significantly at p < 0.05. aindicates significance between UTC group and b indicates significant difference from ISO group.

### 3.2 Histopathological observation

**Fig. 2:**
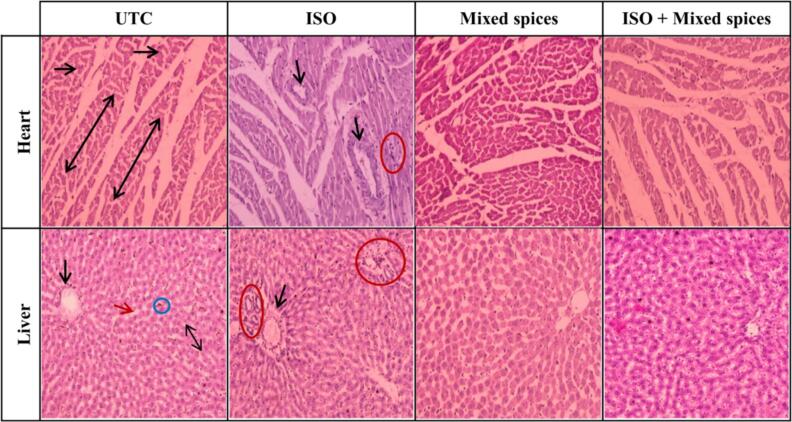
**Histopathology of heart and liver tissues from different groups.** The heart of UTC and Mixed spices group shows regular arrangements of myofibrils (up-down arrow) normal distribution of nuclei (rightwards arrow) (black spots on myofibrils), and no disruption or inflammation in myofibrils. ISO group heart shows disruption of myofibrils (downwards arrow), aggregation of nuclei (red circle) and thus breakage occurs in the normal z lines of myocardial cells. Myofibrilar disruption and nuclei disarrangements are seen rarely or not in ISO + Mixed spices group. UTC and Mixed spices group has classical hepatic architecture. Hepatocytes are arranged in cords radiating from the central vein (black downwards arrow) and separated by blood sinusoids (red arrow), showing hepatocytes polyhydral in shape with cytoplasm and vesicular nuclei. Blood sinusoids (red arrow) separating the hepatic cords are seen lined by endothelial cells (up-down arrow) and kupffer cells (green circle) binucleated hepatocytes are also seen. In ISO group, aggregation of nuclei in sinusoid (red circle) and disruption in central veins (black downwards arrow) is seen while there is no disruption or nuclei aggregation in ISO + Mixed spices group. (For interpretation of the references to color in this figure legend, the reader is referred to the web version of this article.)

**Fig. 3:**
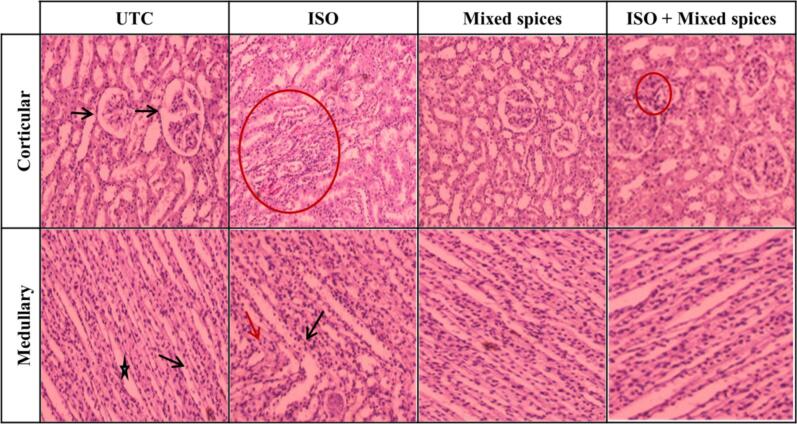
**Histopathology of kidney tissue from different groups.** UTC group’s corticular part of the kidney, renal tubules are lined with typical thick cubic epithelium. The tubules have a relatively regular distinct lumen. Lobular organization of the glomerule and a flat epithelium lining the glomerular capsule (black arrow) can be seen. In modullary part, collecting tubules (black star) are lined with the relatively low simple cubic epithelium. The thick descending and ascending parts of Henle’s loops (black arrow) and a small amount of interstitial tissue can be seen in the cross-sections. A similar architecture is seen in Mixed spices group. In ISO group, some cells of tubular epithelium show features of oedema, capillaries are filled with blood cells (red circle in corticular part). Nuclear aggregation is seen in both collecting tubules (black arrow in medullary part) and Henle’s loops (red arrow in medullary part). This phenomenon was common view in male rats but not in female. No disruption is seen in medullary part of kidney in ISO + Mixed spices group while aggregation of nuclei outside the epithelium cells of glomeruli is rarely seen in corticular part (red circle). (For interpretation of the references to color in this figure legend, the reader is referred to the web version of this article.)

In heart, unvarying dispersal of myofibrils and nuclei and in liver, normal arrangements of central vein, blood sinusoids and hepatocytes with no inflammation or disruption was observed in UTC (Fig. 2) while the kidney of UTC group had typical architecture of glomerular capsule, collecting tubules and Henles’ loops (Fig. 3). Mixed spices group (Fig. 2 and Fig. 3) showed similar organization of tissues from heart, liver and kidney for both male and female rats delineating no adverse effect of spices on these organs.

In ISO group, heart histograph showed disrupted myofibrils, nuclei disarrangements and rupture of z lines besides, liver tissue displayed nuclei-clump and damaged central vein (Fig. 2). Epithelial oedema and nuclei aggregation was found in kidney tissue of ISO group (Fig. 3). All these phenomena of heart, liver and kidney tissue from ISO group indicated pernicious effect of ISO on those tissues.

Rarely or no appearance of disrupted myofibrils and disarranged nuclei in ISO + Mixed spices groups’ heart (Fig. 2) pointed out prevention of MI by mixed spices intake. Both in liver (Fig. 2) and kidney tissue (Fig. 3) displayed rarely or no disruption or clump of nuclei.

### 3.3 Validation of histopathological observation by RT-qPCR

**Fig. 4:**
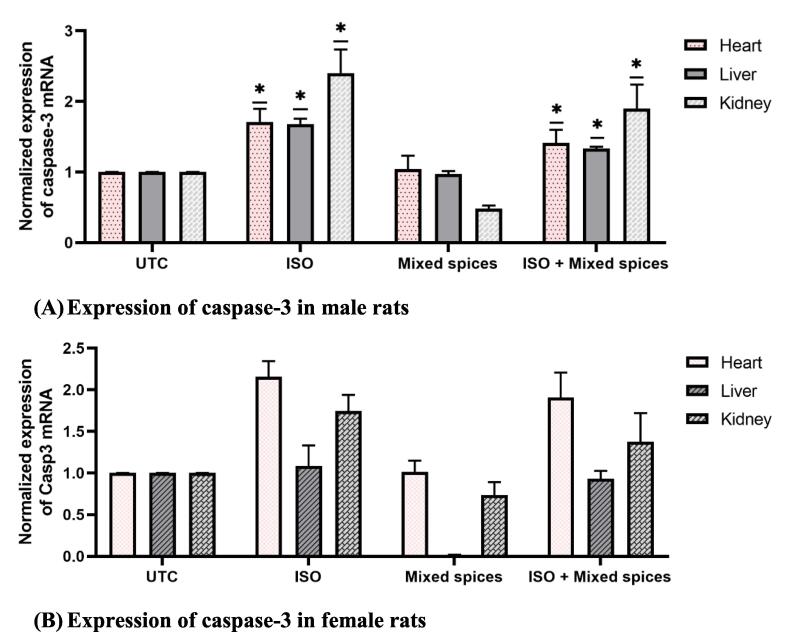
Relative expression of caspase-3 mRNA by RT-qPCR analysis in male (A) and female (B) rats. Caspase-3 mRNA expression was increased in ISO induced rats while ISO + Mixed spices group displayed reduction of caspase-3 gene than ISO group.

Cardiac overexpressing caspase-3 in transgenic mice revealed enlarged infarct size and manifested susceptibility to die after I/R injury while knockdown decreased infarct size and improved heart function in an acute myocardial infarction rat model.^5^, ^19^ Therefore, caspase-3 is one of the important biomarkers involved in myocardial infarction. To validate our histopathological observation in this study we checked the mRNA expression pattern of caspase-3 in heart, kidney, and liver tissues in all groups of male rats. Where we found caspase-3 mRNA expression was elevated after ISO treatment by 71 %, 68 %, and 140 % in heart, kidney, and liver tissues respectively compared to Untreated Control (UTC) (Fig. 4A; UTC and ISO group rats).

Interestingly, mixed spices treatment moderately reduced the caspase-3 expression in all tissues in ISO-induced-MI rats (Fig. 4A; ISO-Mixed spices group in the heart). However, the reduction was not much as the base level of expression (UTC) but decline up to 21 % by mixed spices treatment. Female rats also showed the almost same pattern of caspase-3 expression in heart tissue (Fig. 4B). This indicates mixed spices modulated programmed cell death like apoptotic marker caspase-3 mRNA expression in induced-myocardial infarction rats.

After all, the anthropometric, histopathological, and caspase-3 gene expression analyses results indicated that mixed spices can help in alleviation of myocardial infarction. Other cardiac associated biomarker analysis and protein level experiments are necessary to develop mixed spices as a future medicine for myocardial infarction.

### 3.4 Protein-ligand binding score analysis by molecular docking study

A molecular docking research examined target protein-phytochemicals molecular interactions and binding affinity. Table 2 shows the binding score between caspase-3 (PDB ID: 3KJF) and phytochemicals. After docking, the top three compounds (CID: 95779, CID: 10820, and CID: 6862) were chosen with a cut-off energy of −6.00 kcal/mol. Control drug CID:6167 had a binding score of −4.268 kcal/mol for caspase-3. Through molecular docking, our three compounds outperformed the control drug. So, the final three compounds (CID: 95779, CID:10820, and CID: 6862) were considered for further evaluation in this study.

**Table 2:** A list of various interactions and interacting residues of Caspase-3 protein with the compounds were logged from respective docked complexes.

**CID**	**Docking Score (kcal/mol)**	**Hydrogen bond**	**Others bond**
95,779	−6.112	TRP214, PHE250	TRP206, ARG207, ASN208, PHE247, GLU248, SER249, SER251
10,820	−6.206	TRP214	TRP206, ARG207, ASN208, PHE247, SER249, PHE250, SER251, PHE256
6862	−6.112	SER209	TRP206, ARG207, ASN208, TRP214, GLU248, SER249, PHE250
6167 (caspase-3 inhibitor)	−4.268	N/A	TRP206, ARG207, ASN208, SER209, TRP214, PHE247, GLU248, SER249, PHE250, SER251, PHE252, ASP253, PHE256

### 3.5 Protein-ligand interaction analysis

**Fig. 5:**
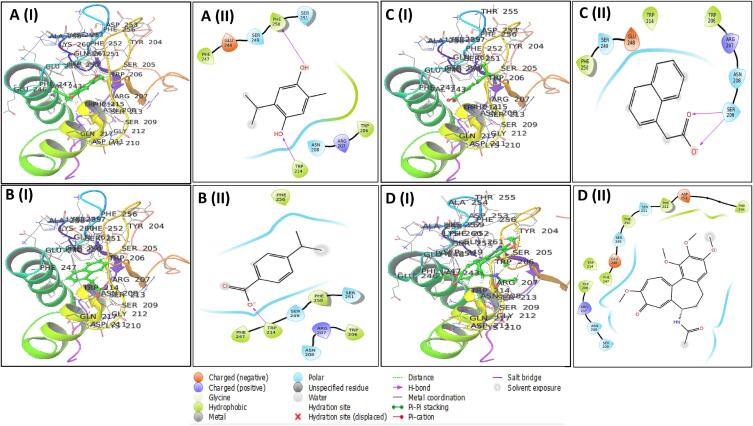
Depicts the molecular interactions between the caspase-3 protein and three specific phytocompounds, as illustrated in 3D and 2D representations. A(I), B(I), C(I) represents 3D representation while A(II), B(II), C (II) shows 2D representation of compounds CID: 95779, CID: 10820, CID: 6862 respectively, additionally, D(I), D(II) represents 3D and 2D respectively for the caspase-3 protein inhibitor complex ligands CID: 6167.

The best docking score comprising three compounds was picked for further analysis, and the Schrodinger suite's Maestro module displayed molecular interactions in Fig. 5 for 3D and 2D structure of protein–ligand complexes. Non-bonded interactions between receptors and ligands include hydrogen, electrostatic, and hydrophobic bonding has been identified. Table 2 shows that CID: 95779, CID: 10820, and CID: 6862 have energy scores of −6.254, −6.206, and −6.112 kcal/mol. Fig. 5 and Table 2 reveal that all three compounds and control medicines interacted with a distinct amino acid. Caspase-3 inhibitors lack hydrogen bonding. Non-covalent bonds are found in TRP206, ARG207, ASN208, SER209, TRP214, PHE247, GLU248, SER249, PHE250, SER251, PHE252, ASP253, PHE256 CID: 95,779 had two hydrogen bonds at TRP214, PHE250 residual position, whereas CID: 10,820 and CID: had one at TRP214 and SER 209 residual places. The caspase-3 inhibitors and three ligands share the TRP206, ARG207, ASN208, and SER249 residues.

### 3.6 Assessment of pharmacokinetics properties and toxicity

Route of administration and bodily organ function affect ADME pharmacokinetics, which rely on patient characteristics and drug chemical properties.^36^ These features may suggest that medications will not fail clinical trials for causes that might have been avoided. The chosen drugs' pharmacokinetics have been assessed. Table 3 lists the compounds (CID: 95779, CID:10820, and CID: 6862) and caspase-3 inhibitor CID:6167 based on their physicochemical, lipophilicity, water-solubility, gastrointestinal absorption, drug-likeness (Lipinski's rule of five), and synthesis accessibility. All substances in the research exhibit favourable pharmacokinetics, indicating low clinical trial failure rates.

**Table 3:** Pharmacokinetics properties include physicochemical properties, lipophilicity, water-solubility, gastrointestinal absorption, drug-likeness, and synthesis accessibility of selected 3 compounds and control drug.

**CID Number**		**CID-6862**	**CID-10820**	**CID-95779**	**CID-6167 (Caspase-3 inhibitor)**
**Physico-chemical Properties**	MW (g/mol)	186.21	164.2	166.22	399.44
	Heavy atoms	14	12	12	29
	Arom. heavy atoms	10	6	6	13
	Rotatable bonds	2	0.3	1	6
	H-bond acceptors	2	2	2	6
	H-bond donors	1	2	2	1
**Lipophilicity**	Log Po/w(MLOGP)	2.65	1	2.1	1.02
**Water Solubility**	Log S (ESOL)	−2.8	2.65	−3.03	−2.9
**Pharmacokinetics**	GI absorption	High	High	High	High
**Drug likeness**	Lipinski, Violation	Yes;0	Yes;0	Yes;0	Yes;0
**Medi. Chemistry**	Synth. accessibility	1.17	1	1.05	3.87
**Mutagenicity & Toxicity**	BBB permeant	Yes	Yes	Yes	No
	Hepatotoxicity(probability)	Active;0.62	Active;0.56	Inactive;0.77	Inactive;0.65
	Carcinogenicity(probability)	Inactive;0.76	Inactive;0.80	Inactive;0.73	Inactive;0.55
	Immunogenicity(probability)	Inactive;0.99	Inactive;0.99	Inactive;0.89	Active;0.99
	Mutagenicity(probability)	Inactive;0.80	Inactive;0.98	Inactive;0.99	Inactive;0.89
	Cytotoxicity(probability)	Inactive;0.86	Inactive;0.86	Inactive;0.90	Active;0.88
	Predicted LD50	670 mg/kg	473 mg/kg	1000 mg/kg	6 mg/kg
	Predicted Toxicity Class	4	4	4	2

Toxicity is a chemical compound's harmfulness to living things. A single or short-term exposure to the toxin might destroy important enzymes and cause significant organ failure. Drug candidates may be hazardous and detrimental to other organs, causing organ toxicity, immunotoxicity, mutagenicity, and cytotoxicity in humans and animals. Therefore, the toxicity of the three substances was also examined in this investigation. Toxicity profiling showed excellent for CID: 95779, CID: 10820, and CID: 6862 with no or low toxicity for human consumption. Three chosen drugs had projected LD50 values of 670 mg/kg, 473 mg/kg, and 1000 mg/kg in toxicity classes 4, 4, and 4, respectively, as shown in Table 3.

### 3.7 ***Interaction of protein***–***ligand stability analysis by molecular dynamic simulation***

Molecular dynamic simulations of the protein–ligand complex structure were used to estimate the stability of the three potential hit phytocompounds (CID: 95779, CID: 10820, and CID: 6862) to the protein's binding site taking into account RMSD, RMSF, rGyr, and SASA to evaluate the chosen caspase-3 protein-phytocompound interaction constancy.

#### 3.7.1 RMSD analysis

**Fig. 6:**
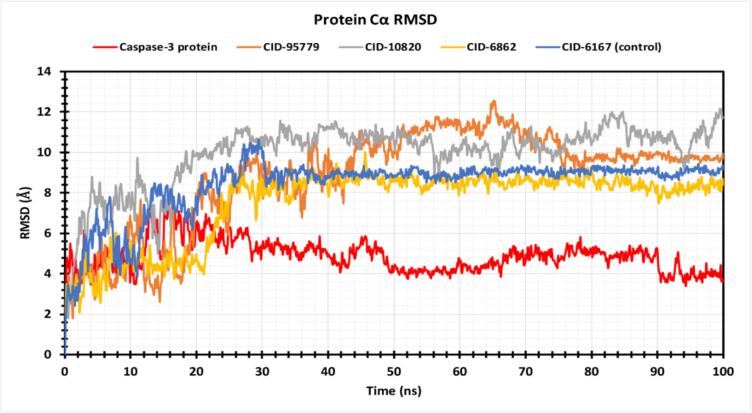
Graphs exhibiting the information about the RMSD values by 100 ns time of MD simulation. The RMSD of caspase-3 protein is shown in red color, where selected compounds are in orange, purple, yellow and blue color respectively. (For interpretation of the references to color in this figure legend, the reader is referred to the web version of this article.)

To assess protein structural stability throughout the 100 ns simulation, the root means square deviation (RMSD) of Cα atoms was subtracted for three hit compounds. According to Fig. 6, the selected phytocompounds CID: 95779, CID: 10820, and CID: 6862 exhibited average fluctuations of 8.79 Å, 9.91 Å, and 7.49 Å, respectively, while the caspase-3 apoprotein and inhibitor ligand complex exhibited 4.87 Å and 8.41 Å, respectively. This suggests that the protein–ligand complex structure is conformationally stable. Some compounds had the least range of variation from the apo but were closer to the capase-3 inhibitor complex, which indicated protein-compound stability by RMSD value. CID:95779, CID:10820, and CID:6862 show high RMSD fluctuations (12.563 Å, 12.156 Å, and 10.023 Å) and low RMSD values (1.824 Å, 1.80 Å, and 2.088 Å). Apoprotein and caspase inhibitor ligand (CID:6167) show good stability (2.113 Å to 7.809 Å and 1.59 Å to 10.647 Å), indicating promising anti-caspase compounds.

#### 3.7.2 RMSF analysis

**Fig. 7:**
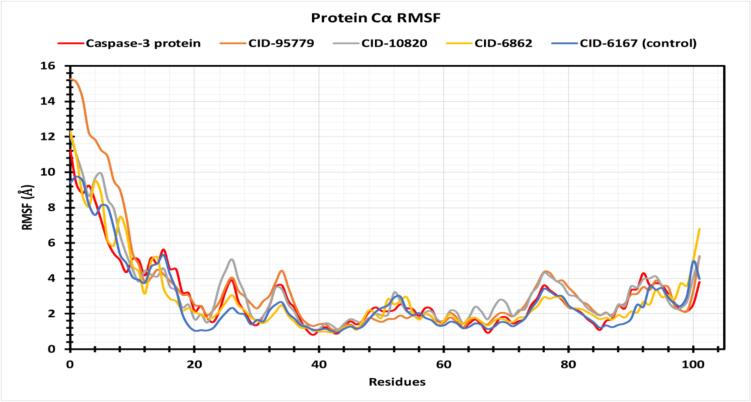
Graphs exhibiting the information about the RMSF values by 100 ns time of MD simulation. The RMSF of caspase-3 protein is shown in red color, where selected compounds are in orange, purple, yellow and blue color respectively. (For interpretation of the references to color in this figure legend, the reader is referred to the web version of this article.)

To study protein structural flexibility after ligand attachment to a certain residue location, the RMSF values of CID: 95779, CID:10820, and CID: 6862 in interaction with caspase-3 were investigated and presented in Fig. 7. Caspase apo protein exhibited the greatest peak fluctuation with ALA 191, GLY 202, LYS 210, PHE 252, and MET 268 at residues 15, 25, 34, 76, and 92, whereas natural ligand had the highest peak fluctuation with MET 182, ALA 191, LYS 229, ASP 253, and TYR 276 at residues 6, 15, 53, 77, Since the protein is N- and C-terminal, the three most significant components had a big peak at the beginning and finish. These three chemicals have acceptable peak ranges. Some typical protein peak sites at ALA 191 amino acid residue positions generate the most simulated change. CID:10820 and CID:6862 had higher RMSF values of 4.11 Å and 4.13 Å at residue 77 of ASP 253. Except for this site, lead compounds fluctuated less than apoprotein and control caspase inhibitor. CIDs 95779, 10820, and 6862 have average RMSF values of 3.496 Å, 3.253 Å, and 2.885 Å. Apoprotein and control medicines had average RMSF values of 2.93 Å and 2.69 Å.

#### 3.7.3 Radius of gyration (Rg)

**Fig. 8:**
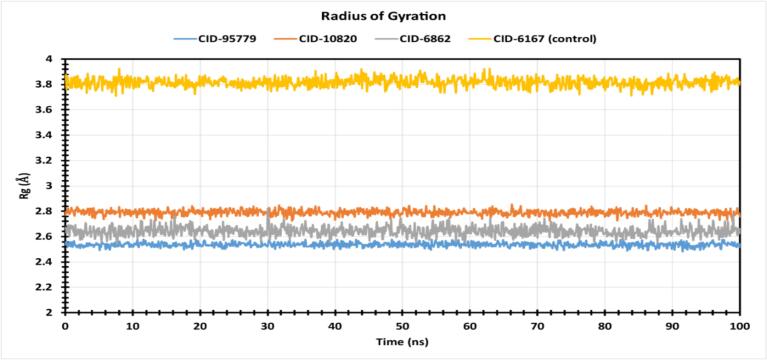
The radius of gyration (Rg) of the protein–ligand complex was calculated from the 100 ns simulation. The Rg value of the selected four compounds CID: 95779, CID: 10820, CID: 6862, and CID: 6167 in complex with the caspase-3 protein represented by a blue, orange, purple, and yellow color respectively. (For interpretation of the references to color in this figure legend, the reader is referred to the web version of this article.)

A protein-compound complex's radius of gyration (Rg) measures atom distribution along its axis. It is a key measure of macromolecule structural activity and complex compactness. As shown in Fig. 8, the stability of CID: 95779, CID:10820, CID: 6862, and the control ligand (CID:6167) in association with the caspase-3 protein was studied using Rg values throughout a 100-ns simulation period. CID:6167 (control) and CID:95779 showed Rg ranges of 3.706 Å to 3.924 Å (fluctuation 0.218 Å) and 2.483 Å to 2.579 Å (fluctuation 0.096 Å), while CID:10820 and CID:6862 showed 2.718 Å to 2.854 Å (fluctuation 0.136 Å) and 2.542 Å to 2.821 Å (fluctuation 0.279 Å). In 100 ns simulations, the three phytocompounds were more stable and had a smaller fluctuation range than control drug complexes, demonstrating that binding did not substantially change the caspase active site conformation.

#### 3.7.4 Solvent accessible surface area (SASA)

**Fig. 9:**
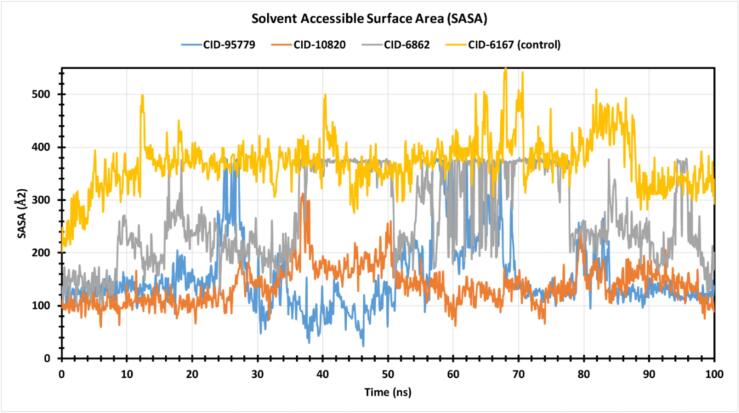
The by solvent accessible surface area (SASA) of the protein–ligand complex was calculated from 150 ns simulation. The SASA value of the selected four compounds CID: 95779, CID: 10820, CID: 6862, and CID: 6167 in complex with the caspase-3 protein represented by a blue, orange, red, and yellow color, respectively. (For interpretation of the references to color in this figure legend, the reader is referred to the web version of this article.)

SASA analysis was done for the phytocompounds to assess the caspase-3 protein and protein–compound complex solvent-like properties (hydrophobic or hydrophilic). Fig. 9 shows that caspase-3 control medications fluctuated between 201.136 Å2 and 555.765 Å2, with an average variation of 370.171 Å2. For phytocompounds CID: 95779, CID: 10820, and CID: 6862, the fluctuation range was 25.018 Å2 to 377.554 Å2, 61.285 Å2 to 311.917 Å2, and 100.92 Å2 to 379.24 Å2, with an average fluctuation of 153.132 Å2, 137.495 Å2, and 265.577 Å2, indicating higher exposure of amino acid residues to the phytocompound in complex systems.

## 4 Discussion

Heart failure (HF) often begins with Myocardial Infarction (MI).^37^ Apoptotic signalling pathway affects MI. One of the main mediators of cardiac tissue apoptosis is caspase-3.^38^ Caspase-mediated pathways, both extrinsic (death receptor involved) and intrinsic (mitochondrial) usually converges on an ultimate effector caspase-3, where caspase-8 and caspase-9 have initial effects. Biochemical and morphological alterations after activation of caspase-3 actually delineates characteristics of myocardial infarction.^39^ Therefore, in the present study we targeted caspase-3. As plant-derived medication options are getting more attractive due to their negligible adverse effects, we used 14 different spices that are used commonly in our everyday life to examine their synergistic molecular effects. We also delve into *in silico* approaches to identify anti-caspase-3 compounds for suggesting herbal remedies.

In wet lab, we prepared mixed spices (aqueous extract) from collected samples and designed the experiment into four groups of rats for treating them for a total of 30 days. We have analysed anthropometric, histologic and molecular data for validation of synergistic effects of spices. Anthropometrically, compared to normal rats, ISO administration increases heart weight and HW/FBW ratio in rats.^40^ Here, ISO increased male and female rat heart weight by 32 % and 30 % compared to UTC. In ISO + Mixed spices group of male and female rats, heart weight was reduced by 29 % and 23 %, respectively (Fig. 1B). In addition, HW/FBW ratio rose by 0.14 (male rats) and 0.19 (female rats) in ISO treated group compared to UTC group, whereas it decreased by 0.12 (male rats) and 0.12 (female rats) in ISO + Mixed spices group compared to ISO treated group. Final BMI was lower in the ISO group but higher in the Mixed spices and ISO + Mixed spices groups owing to, may be, consumed carbohydrate content as thirty days high-carbohydrate diet raised rats’ BMI^41^ and body metabolic changes of animals. So, it can be assumed that Mixed spices reduced BMI, heart weight, and HW/FBW in the ISO + Mixed spices group. Furthermore, biochemical studies of blood sample could provide a potential alignment with this anthropometric research.

ISO-injected animals acquire infarct-like lesions.^42^ Here, histopathology of UTC rats' heart, kidney, and liver tissues exhibited normal architecture, but ISO rats' did not. In the cardiac histology of both male and female rats, the UTC group showed typical myofibril configurations (regular z lines) and nuclei distribution, whereas the ISO group showed extensive disarrangements. The outcome was pertinent to the research of Panda.^43^ Spices treated group had regular cardiac tissue configurations like UTC, whereas ISO + Mixed spices had little or no myofibril disruption (Fig. 2). In kidney histological study, the UTC group of male and female rats had normal kidneys with thick cubic epithelium and a regular lumen. Bowmann's capsule and Henle's loop were likewise normal. In the ISO group, increased glomeruli and tubular epithelial oedema were seen. Liu saw a similar phenomenon.^44^ ISO + Mixed spices group exhibited no fibrosis or a large decrease compared to ISO group (Fig. 3). In liver histophotographs, endothelial cells and kupffer cells border blood sinusoids separating hepatic cords in UTC liver tissue. ISO group had substantial sinusoid nuclei aggregation and central vein rupture, unlike UTC. Hasanzadeh-Moghadam also discovered significant liver fibrosis in ISO-treated rats.^45^ On the other hand, ISO + Mixed spices group exhibited little or no disruption in central veins, like UTC (Fig. 2). As histological study revealed normal arrangements of heart, kidney and liver in ISO + Mixed spices group, it is assumed that mixed spices reduces the detrimental effects of ISO.

Human genetics have revealed apoptotic genes association in MI's possible apoptotic aetiology in recent decades. Caspase and Bcl-2 gene families regulate apoptosis. The Bcl-2 gene family stabilises (Bcl-2-like) or destabilises (Bax-like) the mitochondrial membrane to activate caspases and release cytochrome *c*. After Casp8 activation, Casp3 activation occurs, which is activated by membrane-dependent cues such TNF-α/TNF-αR interaction.^46^ Activation of Casp3 causes myocardial infarction and heart tissue apoptosis. Up-regulation of this Casp3 gene increases myocardial infarct size.^11^, ^46^ In this research, ISO treatment group shows up-regulation of caspase-3 gene, producing MI in cardiac tissue. This study demonstrates that caspase-3 mRNA rose in both kidney and liver in ISO group (Fig. 4), broadening caspase-3 gene's apoptotic activity demonstrated by histomicrophotograph (Fig. 2 and Fig. 3).

Some spices-related therapeutic approaches have already been tried for inhibiting caspase-3 activity. Ginger therapy in rats given 3–4, methylenedioxymethamphetamine (MDMA) decreased caspase-3 mRNA expression, suggesting protection against MDMA-induced cell death.^47^ Garlic extract decreases caspase-3 gene expression in ISO-induced rats.^26^ A recent study has revealed that ISO-administered rats treated with UMB (Umbelliferone) downregulated Casp3 and Bax gene whereas Bcl-2 was upregulated.^48^ In this research, Mixed spices down-regulated Caspase-3 gene expression in kidney (female rats) and liver (male and female rats) compared to UTC (Fig. 4). The histology research showed normal organ tissue structure in this group. This research found no necrosis or inflammation in cardiac, renal, or hepatic tissue in the mixed spices group. In ISO + Mixed spices, caspase-3 expression reduced. In the ISO + Mixed spices group, renal, cardiac, and hepatic tissue showed little or no necrosis and inflammation.

In this study caspase-3 mRNA expression significantly increased in heart, kidney, and liver tissues (71 %, 68 %, and 140 % respectively) following Isoproterenol (ISO) treatment that highlighting the role of apoptosis in myocardial infarction. This aligns with findings that caspase-3 is a critical executor of apoptosis, particularly in cardiac tissues under stress.^76^, ^77^ The mixed spices treatment led to a moderate reduction (21 %) in caspase-3 expression, suggesting potential cardioprotective properties, although not sufficient to restore baseline levels. Similar studies have shown that various natural compounds can modulate caspase-3 activity,^26^, ^78^ indicating a possible therapeutic avenue for myocardial infarction). Female rats exhibited a similar pattern of caspase-3 expression; however, they did not show a significant reduction in expression levels after mixed spice treatment. This suggests that the protective effects of mixed spices may vary across genders, as other studies have also reported gender-specific results in MI.^79^, ^80^, ^81^ While the study highlights the potential of mixed spices in modulating apoptosis, it is important to note that further research is needed to investigate the mechanisms underlying this cardioprotective effect.

For *in silico* analysis, we imported 5027 phytochemicals from 14 spices from IMPPAT server and evaluated through preliminary drug likeness (Lipinski, GI tract permeability) screening and annotated 820 unique compounds for caspase-3. After analysing protein–ligand binding score and interaction, pharmacokinetics, and toxicity, we chose the best tree compounds (CID: 95779, 10820, and 6862) for anti-CASP-3 activity. Previous research articles suggested that when ligands bind with protein more than −5.00 kcal/mol, it shows significantly strong binding of protein-drug complexes.^49^ In our case, CID: 95779, 10820, and 6862 demonstrated favourable binding scores that were more than −6.00 kcal/mol. The pharmacokinetics of all three of the chosen compounds was assessed, and the Lipinski's rules (RO5) for molecules were verified. Good pharmacokinetic characteristics and RO5 maintenance were shown by all of the chosen compounds. To determine the detrimental impact on humans or animals, the chemical with excellent ADME capabilities had been further assessed using its toxicity properties.^50^ Three compounds were chosen, and their levels of toxicity were either non-existent or very low.

The stability of the three possible hit phyto-compounds was then analysed using molecular dynamics simulation. Molecular dynamics simulation confirms protein stability complexed with ligands.^50^ It also measures protein–ligand complex stability and stiffness in a simulated body environment.^51^ RMSD measurements show compound stability, whereas RMSF values reflect mean fluctuation and protein–ligand complex compactness.^29^ Calculating the RMSD of the protein–ligand combination using Cα atoms confirmed little protein fluctuation. Based on the RMSF value, the protein fluctuation was also evaluated. Low complex system fluctuation showed compound stability to the required protein. The protein C and N terminal centre of mass checks protein stability and provides a better knowledge of protein folding for Rg computed.^36^ Lower Rg values indicate strong compactness, whereas higher values indicate compound disassociation with protein, and all compounds have superior Rg values.^29^ Higher SASA values imply a less stable structure, whereas lower values indicate a tightly condensed combination of water molecules and amino acid residues.^29^, ^36^ The research established optimal Rg and SASA values for the selected four compounds. As, our selected compounds has no detrimental effects on animals or humans, showed superior stability and compactness, displayed optimal Rg and SASA values; we can consider our selected compounds as the best alternatives for existing MI-drugs especially Colchicine.

Colchicine (CID: 6167), the pharmacological control for this study, lowers inflammation and infarct size.^52^, ^53^ However, unadjusted colchicine doses, particularly when combined with CYP3A4 inhibitors, have killed chronic renal insufficiency patients. Over 20 % of colchicine users have diarrhea, vomiting, and nausea, whereas myopathy, rhabdomyolysis, and myelosuppression are unusual acute side effects.^54^ Colchicine failed to improve cardiovascular or all-cause mortality, recurrent MI, or other cardiovascular outcomes in post-acute MI patients.^55^ Our proposed phytochemicals may replace colchicine. Among the predicted phytochemicals, Thymohydroquinone (CID: 95779) have strong AChE (acetylcholinesterase) inhibitory properties,^56^ and drug potentiality for treatment of neurological disorders.^57^ Furthermore, thymohydroquinone is nontoxic and has anti-SARS-CoV-2 action.^58^ 4-Isopropylbenzoic acid/cumic acid (CID: 10820) has antifungal properties and shows promise as an inhibitor of SARS-CoV-2 spike and papain-like protease.^59^ 1-naphthaleneacetic acid (CID: 6862) may be used to treat digestive difficulties caused by excessive fat consumption, as well as functional diseases of the gallbladder and bile ducts.^60^ A recent study has revealed that spices compound- gingerol, curcumin, and capsaicin are potential COX/5-LOX inhibitors, promising anti-inflammatory agents.^61^ Therapeutic study of fourteen spices used in this study has potentiality to treat different types of cardiovascular disorders (Supplementary file-4). Our *in silico* study has marked up thymohydroquinone (CID: 95779) as the best lead compound for anti-caspase-3 drug potentiality due to its low toxicity than other two compounds (CID: 10,820 and CID: 6862). So, this compound can be used for drug designing for MI. Although we identified thymohydroquinone as a potential drug candidate, the perfect combination of compounds needs to be studied through different hybrid analytical techniques.

## 5 Conclusion

In summary, our research demonstrates that the water extract of mixed spices offers significant protection against heart injury in rats. This protective effect was validated by improvements in heart size, tissue structure integrity, and a reduction in Caspase-3 mRNA expression, indicating that the combined spices work synergistically to mitigate cardiac damage. Furthermore, computational analysis of the bioactive compounds from the mixed spices identified thymohydroquinone as having the strongest binding affinity to the CASP-3 protein and the lowest predicted toxicity. These findings underscore the potential of mixed spices—particularly thymohydroquinone—as a foundation for developing novel heart-protective therapies targeting apoptotic pathways. However, further studies involving additional animal models and clinical trials are essential to confirm the therapeutic efficacy and safety of thymohydroquinone.

## Ethics statement

Ethical approval of this study was obtained from BTRI, BCSIR, Dhaka-1205, Bangladesh. Ethical clearance reference no: 39.309.006.00.00.163.2014/403 (2021–2).

## Consent for publication

Not applicable.

## Availability of data and materials

Data and materials used in this investigation are accessible from the corresponding author.

## Funding

Bangladesh Council of Scientific and Industrial Research (BCSIR), Dhaka, Bangladesh, financed this research. Funders had no involvement in research design, data collection and analysis, publication decision, or manuscript writing.

## CRediT authorship contribution statement

**Md. Abdullah-Al-Mamun:** Writing – review & editing, Writing – original draft, Resources, Methodology, Investigation, Formal analysis, Data curation, Conceptualization. **Dipa Islam:** Writing – review & editing, Supervision, Resources, Methodology, Funding acquisition, Formal analysis, Data curation, Conceptualization. **Dipankar Chandra Roy:** Writing – original draft, Formal analysis. **Ayesha Ashraf:** Writing – review & editing, Validation. **Saifullah:** Writing – review & editing, Formal analysis. **Chadni Lyzu:** Validation. **Md. Enamul Kabir Talukder:** Writing – review & editing, Software. **Samina Akhter:** Validation. **Evana Parvin Lipy:** Visualization. **Liton Chandra Mohanta:** Visualization.

## Declaration of competing interest

The authors declare that they have no known competing financial interests or personal relationships that could have appeared to influence the work reported in this paper.

## References

[1] Asif M., Bhat S., Nizamuddin S., Mustak M. (2018). Association between myocardial infarction and dermatoglyphics: a cross-sectional study. J Cardiovasc Dis Res..

[2] Mil J.W.F. (2007). Concise clinical pharmacology. Pharmacy World & Science.

[3] Hoda S.A., Hoda R.S. (2020). Robbins and cotran pathologic basis of disease. American Journal of Clinical Pathology.

[4] Topol E.J., Smith J., Plow E.F., Wang Q.K. (2006). Genetic susceptibility to myocardial infarction and coronary artery disease. Human Molecular Genetics.

[5] Mockrin S.C. (1996).

[6] Kariz S. (2012). Genetic markers of myocardial infarction. J Clin Ex Cardiol..

[7] Hadian K., Stockwell B.R. (2023). The therapeutic potential of targeting regulated non-apoptotic cell death. Nat Rev Drug Dis..

[8] Beere M, Green R. Immunologic Repercussions of Cell Death (Chapter 28). In: Kelley and Firestein’s Textbook of Rheumatology. 10th ed. Elsevier; 2017:418-44doi:10.1016/B978-0-323-31696-5.00028-0.

[9] Shalini S., Dorstyn L., Dawar S., Kumar S. (2014). Old, new and emerging functions of caspases. Cell Death and Differentiation.

[10] Moorjani N., Ahmad M., Catarino P. (2006). Activation of apoptotic caspase cascade during the transition to pressure overload-induced heart failure. Journal of the American College of Cardiology.

[11] Condorelli G., Roncarati R., Ross J. (2001). Heart-targeted overexpression of caspase3 in mice increases infarct size and depresses cardiac function. Proceedings of the National Academy of Sciences.

[12] Liu C., Huang Y. (2016). Chinese herbal medicine on cardiovascular diseases and the mechanisms of action. Frontiers in Pharmacology.

[13] Gabisonia K., Prosdocimo G., Aquaro G.D. (2019). MicroRNA therapy stimulates uncontrolled cardiac repair after myocardial infarction in pigs. Nature.

[14] Khurana S., Venkataraman K., Hollingsworth A., Piche M., Tai T. (2013). Polyphenols: benefits to the cardiovascular system in health and in aging. Nutrients.

[15] Matsutomo T. Potential benefits of garlic and other dietary supplements for the management of hypertension (Review). Exp Ther Med. Published online December 27, 2019. doi:10.3892/etm.2019.8375. 10.3892/etm.2019.8375 PMC6966105 32010326

[16] Shaito A., Thuan D.T.B., Phu H.T. (2020). Herbal medicine for cardiovascular diseases: efficacy, mechanisms, and safety. Frontiers in Pharmacology.

[17] Pandey S., Singh B.K. (2019). De-novo drug design, molecular docking and in-silico molecular prediction of AChEI analogues through CADD approaches as Anti-Alzheimer’s agents. Current Computer-Aided Drug Design.

[18] Rahim F., Putra P.P., Ismed F., Putra A.E., Lucida H. (2023). Molecular dynamics, docking and prediction of absorption, distribution, metabolism and excretion of lycopene as protein inhibitor of Bcl2 and DNMT1. Trop J Nat Product Res..

[19] Modanwal S., Mulpuru V., Mishra N. (2023). Structure-guided design and optimization of small molecules as pancreatic lipase inhibitors using pharmacophore, 3D-QSAR, molecular docking, and molecular dynamics simulation studies. Current Computer-Aided Drug Design.

[20] Şahin S., Dege N. (2021). A newly synthesized small molecule: the evaluation against Alzheimer’s disease by in silico drug design and computational structure analysis methods. Journal of Molecular Structure.

[21] Parham J.S., Goldberg A.C. (2022). Review of recent clinical trials and their impact on the treatment of hypercholesterolemia. Progress in Cardiovascular Diseases.

[22] Test No. 407: Repeated Dose 28-day Oral Toxicity Study in Rodents.; 2008. doi:10.1787/9789264070684-en.

[23] García CMR, Echeverría LLP. Preparation of aqueous extracts of plants v1. protocols.io. Published online August 27, 2018. doi:10.17504/protocols.io.szwef7e.

[24] Sun S.J., Wu X.P., Song H.L., Li G.Q. (2015). Baicalin ameliorates isoproterenol-induced acute myocardial infarction through iNOS, inflammation, oxidative stress and P38MAPK pathway in rat. Int J Clin Experimen Med..

[25] Saifullah N., Sakari M., Suzuki T., Yano S., Tsukahara T. (2020). Effective RNA knockdown using CRISPR-Cas13a and molecular targeting of the EML4-ALK transcript in H3122 lung cancer cells. International Journal of Molecular Sciences.

[26] Islam D., Shanta M.B., Akhter S. (2020). Cardioprotective effect of garlic extract in isoproterenol-induced myocardial infarction in a rat model: assessment of pro-apoptotic caspase-3 gene expression. Clinical Phytoscience.

[27] Livak K.J., Schmittgen T.D. (2001). Analysis of relative gene expression data using real-time quantitative PCR and the 2−ΔΔCT method. Methods.

[28] Saifullah N., Tsukahara T. (2022). Integrated analysis of ALK higher expression in human cancer and downregulation in LUAD using RNA molecular scissors. Clinical & Translational Oncology.

[29] Ahammad F., Alam R., Mahmud R. (2021). Pharmacoinformatics and molecular dynamics simulation-based phytochemical screening of neem plant (Azadiractha indica) against human cancer by targeting MCM7 protein. Briefings in Bioinformatics.

[30] Vivek-Ananth R.P., Mohanraj K., Sahoo A.K., Samal A. (2023). IMPPAT 2.0: an enhanced and expanded phytochemical atlas of indian medicinal plants. ACS Omega.

[31] Halgren T. (2007). New method for fast and accurate binding‐site identification and analysis. Chemical Biology & Drug Design.

[32] Harder E., Damm W., Maple J. (2015). OPLS3: a force field providing broad coverage of drug-like small molecules and proteins. Journal of Chemical Theory and Computation.

[33] Friesner R.A., Murphy R.B., Repasky M.P. (2006). Extra precision glide: docking and scoring incorporating a model of hydrophobic enclosure for protein−ligand complexes. Journal of Medicinal Chemistry.

[34] Daina A., Michielin O., Zoete V. (2017). SwissADME: a free web tool to evaluate pharmacokinetics, drug-likeness and medicinal chemistry friendliness of small molecules. Scientific Reports.

[35] Banerjee P., Eckert A.O., Schrey A.K., Preissner R. (2018). ProTox-II: a webserver for the prediction of toxicity of chemicals. Nucl Acids Res..

[36] Imon R.R., Talukder M.E.K., Akhter S. (2023). Natural defense against multi-drug resistant Pseudomonas aeruginosa: Cassia occidentalis L. in vitro and in silico antibacterial activity. RSC Advances.

[37] Girerd N. (2022). The growing importance of heart failure in myocardial infarction. International Journal of Cardiology.

[38] Zhang Q., Wang L., Wang S. (2022). Signaling pathways and targeted therapy for myocardial infarction. Signal Transduction and Targeted Therapy.

[39] Kim N.H., Kang P.M. (2010). Apoptosis in cardiovascular diseases: mechanism and clinical implications. Korean Circ J..

[40] Li H., Xie Y.H., Yang Q. (2012). Cardioprotective effect of paeonol and danshensu combination on isoproterenol-induced myocardial injury in rats. PLoS One1.

[41] Novelli E.L.B., Diniz Y.S., Galhardi C.M. (2007). Anthropometrical parameters and markers of obesity in rats. Labor Animals..

[42] Khalil M.I., Ahmmed I., Ahmed R. (2015). Amelioration of isoproterenol-induced oxidative damage in rat myocardium by withania somnifera leaf extract. Biomed Research International.

[43] Panda S., Kar A., Biswas S. (2017). Preventive effect of Agnucastoside C against Isoproterenol-induced myocardial injury. Scientific Reports.

[44] Liu Q., Zhang Q., Wang K. (2015). Renal denervation findings on cardiac and renal fibrosis in rats with isoproterenol induced cardiomyopathy. Scientific Reports.

[45] Hasanzadeh-Moghadam M., Khadem-Ansari M.H., Farjah G.H., Rasmi Y. (2018). Hepatoprotective effects of betaine on liver damages followed by myocardial infarction. Vet Res Forum..

[46] Ashkenazi A., Dixit V.M. (1998). Death receptors: signaling and modulation. Science.

[47] Asl S., Pourheydar D., Nezhadi R., Mehdizadeh (2013). Ecstasy-Induced caspase expression alters following ginger treatment. Basic Clin Neurosci..

[48] Althunibat O.Y., Abduh M.S., Abukhalil M.H. (2022). Umbelliferone prevents isoproterenol-induced myocardial injury by upregulating Nrf2/HO-1 signaling, and attenuating oxidative stress, inflammation, and cell death in rats. Biomedicine & Pharmacotherapy.

[49] Talukder M.E.K., Atif M.F., Siddiquee N.H. (2025). Molecular docking, QSAR, and simulation analyses of EGFR-targeting phytochemicals in non-small cell lung cancer. Journal of Molecular Structure.

[50] Aljahdali M.O., Molla M.H.R., Ahammad F. (2021). Compounds identified from marine mangrove plant (avicennia alba) as potential antiviral drug candidates against WDSV, an in-silico approach. Marine Drugs.

[51] Bharadwaj S., Dubey A., Yadava U., Mishra S.K., Kang S.G., Dwivedi V.D. (2020). Exploration of natural compounds with anti-SARS-CoV-2 activity via inhibition of SARS-CoV-2 Mpro. Briefings in Bioinformatics.

[52] Akodad M., Sicard P., Fauconnier J., Roubille F. (2020). Colchicine and myocardial infarction: a review. Archives of Cardiovascular Diseases.

[53] Lazaros G., Imazio M., Brucato A. (2018). The role of colchicine in pericardial syndromes. Current Pharmaceutical Design.

[54] Slobodnick A, Shah B, Krasnokutsky S, Pillinger MH. Update on colchicine, 2017. Rheumatology. 2017;57(suppl_1):i4-i11. doi:10.1093/rheumatology/kex453. 10.1093/rheumatology/kex453 PMC5850858 29272515

[55] Diaz-Arocutipa C., Benites-Meza J.K., Chambergo-Michilot D. (2021). Efficacy and safety of colchicine in post–acute myocardial infarction patients: a systematic review and meta-analysis of randomized controlled trials. Frontiers in Cardiovascular Medicine.

[56] Jukic M., Politeo O., Maksimovic M., Milos M., Milos M. (2006). In Vitro acetylcholinesterase inhibitory properties of thymol, carvacrol and their derivatives thymoquinone and thymohydroquinone. Phytotherapy Research.

[57] Shah F.H., Salman S., Idrees J., Idrees F., Akbar M.Y. (2020). In silico study of thymohydroquinone interaction with blood–brain barrier disrupting proteins. Future Science OA.

[58] Esharkawy E.R., Almalki F., Hadda T.B. (2022). In vitro potential antiviral SARS-CoV-19- activity of natural product thymohydroquinone and dithymoquinone from Nigella sativa. Bioorganic Chemistry.

[59] Patowary L., Borthakur M. (2022). Computational studies of Bridelia retusa phytochemicals for the identification of promising molecules with inhibitory potential against the spike protein and papain-like protease of SARS-CoV-2. Sci Phytochem..

[60] 1-naphthaleneacetic acid. DrugBank Online. Published 2023. Accessed February 23, 2023. https://go.drugbank.com/drugs/DB01750.

[61] Rudrapal M., Eltayeb W.A., Rakshit G. (2023). Dual synergistic inhibition of COX and LOX by potential chemicals from Indian daily spices investigated through detailed computational studies. Scientific Reports.

[62] Kawase Y., Sunagawa Y., Shimizu K. (2023). 6-shogaol, an active component of ginger, inhibits p300 histone acetyltransferase activity and attenuates the development of pressure-overload-induced heart failure. Nutrients.

[63] Anwar S., Bhandari U., Panda B.P. (2018). Trigonelline inhibits intestinal microbial metabolism of choline and its associated cardiovascular risk. Journal of Pharmaceutical and Biomedical Analysis.

[64] Al-Asoom L.I., Al-Shaikh B.A., Bamosa A.O., El-Bahai M.N. (2014). Effect of Nigella sativa supplementation to exercise training in a novel model of physiological cardiac hypertrophy. Cardiovascular Toxicology.

[65] Chouhan H., Purohit A. (2018). Protective effect of cumin (Cuminum cyminum L.) seed extract on cardiovascular system, toxicity, and hematology on hyperlipidemic rabbits: an experimental study. Asian J Pharm Clin Res..

[66] Dhyani N., Parveen A., Siddiqi A., Hussain M.E., Fahim M. (2020). Cardioprotective efficacy of coriandrum sativum (L.) seed extract in heart failure rats through modulation of endothelin receptors and antioxidant potential. J Diet Suppl..

[67] Gazia MA, & El-Magd MA. (2018). Ameliorative Effect of Cardamom Aqueous Extract on Doxorubicin-Induced Cardiotoxicity in Rats. Cells, tissues, organs. 2018;206(1-2):62–72. doi: 10.1159/000496109. 10.1159/000496109 30716735

[68] Malik A., Mehmood M.H., Channa H., Akhtar M.S., Gilani A.H. (2017). Pharmacological basis for the medicinal use of polyherbal formulation and its ingredients in cardiovascular disorders using rodents. BMC Complement Altern Med Ther..

[69] Lu Q.-Y., Rasmussen A.M., Yang J. (2019). Mixed spices at culinary doses have prebiotic effects in healthy adults: a pilot study. Nutrients.

[70] Petersen K.S., Davis K.M., Rogers C.J. (2021). Herbs and spices at a relatively high culinary dosage improves 24-hour ambulatory blood pressure in adults at risk of cardiometabolic diseases: a randomized, crossover, controlled-feeding study. The American Journal of Clinical Nutrition.

[71] Annou G., El Hadj-Khelil A.O. (2021). Phenolic compounds, antioxidant and antibacterial activities of spices mixtures used by population of ouargla. Pharmacophore..

[72] Vemuri S.K., Banala R.R., Subbaiah G.P., Srivastava S.K., Reddy A.G., Malarvili T. (2017). Anti-cancer potential of a mix of natural extracts of turmeric, ginger and garlic: a cell-based study. Egypt J Basic Appl Sci..

[73] Platel K., Rao A., Saraswathi G., Srinivasan K. (2002). Digestive stimulant action of three Indian spice mixes in experimental rats. Food/Nahrung.

[74] Siddiq I.A., Martin O., Lawal N., Muhammad M.B., Usman I.N. (2022). Cardio-and-Hepatoprotective Benefits of Some Spices in Wistar Rats Induced with Metabolic Syndrome. Tropic. J Nat Prod Res..

[75] Islam D., Akter F., Akhter S. (2025). Characterization of sweet potato flakes enriched with chia seeds: nutritional profile, bioactive compounds, sensory attributes, and cardioprotective potential. J Agri Food Res..

[76] Voroshilova T., Shepitko V., Stetsuk E. (2024). Activity of caspase-3 in the interstitial tissues of the ventricular myocardial cells during long-term blockage of release hormone in male rats on the background of quercetine administration. Current Problems of Modern Medicine: Bulletin of the Ukrainian Medical and Dental Academy..

[77] Liu Q. (2014). Lentivirus mediated interference of Caspase-3 expression ameliorates the heart function on rats with acute myocardial infarction. European Review for Medical and Pharmacological Sciences.

[78] Mohammed M.M., Osman N.A., Mourad F.M., Abedelbaky M.F. (2023). Cardioprotective effect of ginger in a rat model of myocardial damage and its possible intervention in perk-atf4-Chop-Puma apoptotic pathway. Ukrainian Biochemical Journal.

[79] Sofia R.R., Serra A.J., Silva J.A. (2014). Gender-based differences in cardiac remodeling and ILK expression after myocardial infarction. Arquivos Brasileiros de Cardiologia.

[80] Komici K., Conti V., Davinelli S. (2020). Cardioprotective effects of dietary phytochemicals on oxidative stress in heart failure by a sex-gender-oriented point of view. Oxidative Medicine and Cellular Longevity.

[81] Pathak K., Pathak M.P., Saikia R. (2024). Cardioprotective activities of some indian Spices: an insight into pharmacology and phytochemical investigation. Curr Tradit Med..

